# Computational design of immunogenic peptide–ligand conjugates for targeted therapy against Nipah virus infection

**DOI:** 10.3389/fbinf.2026.1846380

**Published:** 2026-07-27

**Authors:** Sudip Prasad Jena, Prateek Nayak, M. A. Adithyan, Manoviraj Morey, R. Vihaas Tharsini, Abilash Valsala Gopalakrishnan, Kuntal Pal, Sabina Evan Prince

**Affiliations:** 1 Department of Biotechnology, School of Biosciences and Technology, VIT University, Vellore, Tamil Nadu, India; 2 Department of Bio-Sciences, School of Bio Sciences and Technology (SBST), Vellore Institute of Technology, Vellore, Tamil Nadu, India; 3 Department of Biomedical Sciences, School of Bio-Sciences and Technology, Vellore Institute of Technology, Vellore, Tamil Nadu, India

**Keywords:** immunotherapeutic, *in silico* drug designing, Nipah glycoprotein, Nipah virus, peptide–ligand conjugation

## Abstract

**Introduction:**

Nipah virus (NiV) poses a major risk to global public health due to its high infectivity and associated mortality rates. Currently, no licensed vaccines or antiviral medications are available for NiV infection, leaving clinical management limited to supportive care. The viral receptor glycoprotein responsible for binding NiV to host cell receptors (ephrin-B2/B3) represents an ideal therapeutic target. This study proposes a novel peptide-ligand conjugate (PLC) immunotherapeutic approach that exploits pre-existing immune responses in NiV-endemic populations to selectively target and eliminate infected cells.

**Methods:**

We employed biomolecular modeling (*in silico*) to establish binding affinities and perform docking studies using a compound library obtained from the MolProphet database. A non-cleavable oxime linker was selected to enhance physical stability and ensure robust conjugation between ligand and peptide components. The peptide was engineered to contain immunogenic minimal epitope regions derived from measles, mumps, and rubella vaccines, selected based on their high immunization rates and long-lived memory responses in individuals residing in NiV-endemic areas.

**Results:**

The PLC design demonstrated selective binding capacity to a transmembrane protein present on NiV-infected cells. The oxime linker provided enhanced stability, and the peptide epitope design successfully incorporated regions associated with established long-term immunity.

**Discussion:**

This PLC system represents a promising framework for antiviral therapeutic development by harnessing pre-existing immune recognition to promote selective clearance of NiV-infected cells. The findings highlight critical structural components and functional roles of PLCs in therapeutic development, including drug target screening and rational design strategies for enhancing targeting specificity and molecular stability. Future work should focus on experimental validation of the computational predictions and *in vitro*/*in vivo* efficacy studies.

## Graphical Abstract

**Figure:**
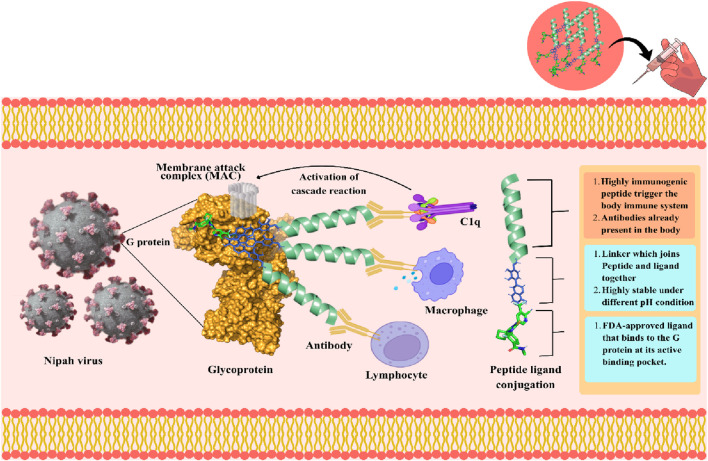

## 1 Introduction

The Nipah virus (NiV) is classified as a zoonotic pathogen that is transmitted from animal reservoirs to human hosts and may also propagate through the consumption of contaminated alimentary products or via direct human-to-human contact (Skowron et al., 2022). The inaugural documented outbreak of the NiV transpired in Malaysia in the year 1998, subsequently followed by an outbreak in Singapore in 1999 (Centers for Disease Control and Prevention, 1999). From that initial occurrence onward, the Southeast and South Asian regions have encountered sporadic outbreaks of NiV infection, documented mainly in Bangladesh, India, and the Philippines (World Health Organization, 2023). The NiV occurrences have predominantly affected individuals who interacted closely with infected intermediate hosts or consumed contaminated date palm sap, which may have been contaminated by bat excretions or saliva (Faus-Cotino et al., 2024). For the second occasion this year, India has documented a lethal case of the NiV, this instance, involving a 24-year-old student, as reported by a local health official to Reuters. The onset of the man’s symptoms occurred on September 4, and he succumbed five days later, as per a district health official from Malappuram, which has been identified as one of the nation’s endemic regions for NiV and where the previous case in the country was detected earlier this year (Schnirring, 2024). Unfortunately, there is no antiviral treatment or vaccination available for the treatment of NiV (Kiran et al., 2025). The 18.2-kb RNA genome of NiV encodes three structural proteins, and the P gene encodes three accessory proteins (Sun et al., 2018). The structural G protein has been a key player in NiV immune modulation, viral replication, assembly, and interaction with host proteins.

Generally, monoclonal antibody-based therapies and traditional vaccination strategies using attenuated viruses and recombinant protein antigens are time-consuming and highly expensive procedures, and the chances of success are low (Cid and Bolívar, 2021). This research is aimed at designing a peptide–ligand conjugate (PLC) for the treatment of NiV infection with minimal toxicity while inducing a strong humoral immune response characterized by a high antibody titer. Since infancy, vaccination has played a crucial role in conferring long-term immunity against numerous infectious diseases. These vaccines function by stimulating the production of pathogen-specific antibodies that efficiently neutralize invading pathogens upon infection, while exhibiting minimal or no cross-reactivity with host cells. The reason we have selected measles, mumps, and rubella (MMR) vaccines for our peptide epitope selection is due to the widespread use of MMR among the populations of the countries where NiV is endemic (World Health Organization, 2019). The protein candidates from the MMR vaccines have long-lasting/lifelong protective antibody titers (Sowers et al., 2016).

We selected a peptide length of 14–20 amino acids, which is enough to induce an immune-mediated response. Furthermore, we analyzed their physicochemical properties, allergenicity, and toxicity values using various in-silico tools. The second essential component of PLC designing is the ligand. We chose an FDA-approved ligand from the MolProphet™ (https://app.molprophet.com/). When designing PLCs, it is crucial to carefully select stable, non-cleavable linkers to prevent cleavage of the peptide and ligand in the bloodstream.

## 2 Materials and methods

### 2.1 Identification of minimal epitope region used in the NiV endemic countries

According to vaccination data from the World Health Organization (World Health Organization, 2026), the MMR vaccine is widely administered in regions where NiV infection is prevalent. Studies have shown that the MMR vaccine can confer long-term immunity (Shah et al., 2024). Immunogenic peptides from MMR toxins are used to make PLCs. These peptides can induce the pre-existing immunity, which can non-specifically target the G protein; they help to clear the G protein through a series of immune-mediated mechanisms. Kleine et al. (1990). determined that the requisite minimum length of peptide sequences for the induction of effective antibody production is 15–20 amino acids. This activates the immune memory B cells for the antibody surge (Inamine et al., 2005). Moreover, prior studies have demonstrated that chemically synthesized peptides as short as 12 amino acids can effectively induce memory antibody production (Tam, 1988). These understandings collectively support the strategic choice of peptide segments spanning 10–20 amino acids, supporting their sustainability for generating strong and sustained memory immune responses (Hamley, 2017).

### 2.2 Predicting the minimal epitope regions from the SVM trip server

We conducted a screening of the peptides present in the MMR vaccine. We performed an *in silico* analysis using the SVM trip server (Yao et al., 2012) that searches through all possible sequences from an entire protein for highly immunogenic regions (stretches of the peptides). After completing this analysis on the entire length of the protein, the server suggests which portion of the entire protein corresponds to an epitope based on how highly immunogenic the stretch of peptides is according to the IEDB EpiMatrix database. The EpiMatrix reference score of the epitope we selected, according to IEDB, was used to determine its immunogenicity, with a score of 0.9–1 being classified as a strongly immunogenic epitope (Yao et al., 2012).

### 2.3 Physicochemical characterization, immunogenicity assessment, and T-cell epitope selection of minimal epitope regions

We further investigated the physicochemical characteristics of the minimal epitope region (MER) using ProtParam (https://web.expasy.org/protparam/). The ProtParam server provides several physical properties that can be derived from a protein sequence, including molecular mass, predicted pI, amino acid content, atomic content, extinction coefficient, half-life, instability index, aliphatic index, and GRAVY. Thus, molecular weight and theoretical pI are determined using the Compute pI/Mw method. ToxinPred (Gupta et al., 2013), AllerTOP (Dimitrov et al., 2014), and VaxiJen (Doytchinova and Flower, 2007) were all used to determine whether the MER may be toxic, allergenic, or antigenic. Each of these servers uses a different algorithm to determine a toxicity or allergenicity score that indicates the likelihood of a protein having those properties. For T-cell epitope selection, we used NetMHC 4.0 (Andreatta and Nielsen, 2016) to predict the binding affinity of peptides to MHC Class I molecules, which helps identify potential CD8^+^ T-cell epitopes. The NetMHC2.3 (Jensen et al., 2018) tool is used to predict peptide binding to MHC class II molecules, which are essential for activating CD4^+^ T-helper cells**.**

### 2.4 Protein and ligand preparation

The solved structure of the glycoprotein of NiV was fetched from the PDB (Protein Data Bank) (PDB ID: 7TXZ) (Wang, 2022). Preparation steps included the removal of all heteroatoms (water molecules, ions, and original ligands), the addition of polar hydrogen atoms, and the assignment of partial charges. The prepared structure was subsequently energy minimized using the AMBER force field to relieve steric clashes and saved in PDB format for docking (Case et al., 2023). A library of 50 therapeutic ligands was curated for this study. These ligands were identified from the MolProphet (https://app.molprophet.com/?q=&p=1), which has FDA-approved drugs. The 3D structures of these ligands were downloaded from PubChem (Kim et al., 2016) and further prepared using the Open Babel toolkit integrated into AutoDock Vina software. Preparation included energy minimization using the universal force field, protonation to assign correct ionization states at physiological pH (7.4), and conversion into the AutoDock-compatible PDBQT format.

### 2.5 Estimation of the ADME profile of the screened compounds

The pharmacokinetic properties of the selected compounds were evaluated using the ADMET (Absorption, Distribution, Metabolism, Excretion, and Toxicity) 3.0 (https://admetlab3.scbdd.com/) server to assess their drug-likeness, absorption, distribution, metabolism, and toxicity profiles. The key parameters analyzed included molecular weight (MW), lipophilicity (LogP), Lipinski rule compliance, blood–brain barrier (BBB) permeability, CYP3A4 inhibition potential, hERG toxicity risk, human liver microsomal (HLM) stability, and predicted half-life (*T*_1/2_).

### 2.6 Molecular docking and binding affinity estimation

Molecular docking was conducted to forecast the binding modes and affinities of the 50 immunogenic ligands with the synthesized NiV G protein. We used AutoDock Vina to run the docking (Eberhardt et al., 2021). To make sure that the conformational space was sampled well, the exhaustiveness parameter was set to 8. We generated and ranked the top 10 conformational poses for each ligand based on how strongly they were predicted to bind (in kcal/mol). A lower Vina score means a stronger binding affinity.

### 2.7 Analysis of protein–ligand interactions and complex selection

The resulting docking poses for the top-scoring ligands were analyzed within AutoDock Vina. The protein–ligand complex with the strongest predicted binding affinity was selected for further epitope analysis. Interactions within this complex were visualized using PyMOL and LigPlot + software (Agrawal et al., 2024).

### 2.8 Selection of linker for ligand-minimal epitope conjugation

**FIGURE 1:**
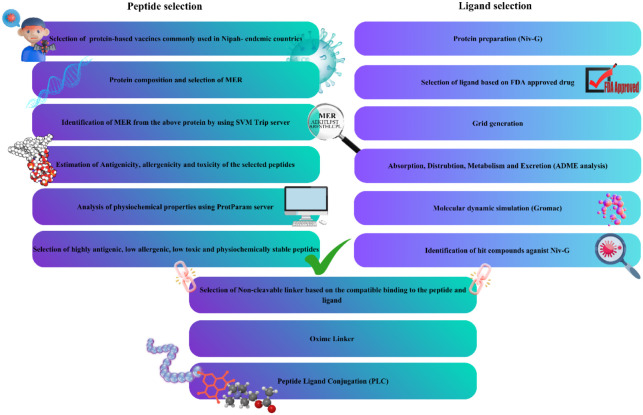
Schematic workflow of PLC designing.

To ensure effective delivery of PLCs, the chosen MER must be linked to the targeting ligand through a stable linker. A suitable non-cleavable linker (such as oxime, triazole, or succinimidyl thioester) was chosen to stop the immunogenic ligand and the N-terminal chemistry of the selected MER from breaking apart too soon in the bloodstream. Among the three linkers, we used the oxime linker because it connects a carbonyl group on the ligand to an amide bond to the peptide. The work flow of PLC designing has been shown in Figure 1 (Mező et al., 2024).

### 2.9 PLC (Peptide Ligand Conjugate) construction and optimization

The PLC design is an intricate task for the assessment of peptide viability upon conjugation with the druggable compound. For performing this framework construction, a combination of server-based tools and locally installed aids was utilized. For the prediction of the peptides’ 3D conformations, PePfold 3 was used (Lamiable et al., 2016). This server-based platform employs a coarse-grained energy function-based method combined with a Markov-based model for swift prediction of conformational space for short peptides (Lamiable et al., 2016). For our study, the top-ranked model was selected based on the lowest sOPEP energy score. Furthermore, the C-terminus was checked to ensure it carried a free carboxyl terminus, and the N terminus was left unblocked to serve as a conjugation handle for subsequent oxime ligation.

Following this, the small molecule ligand was retrieved from the PubChem repository in three-dimensional SDF format; the retrieved structure encodes the full pharmacophore framework of the molecule (National Center for Biotechnology Information, 2021). Furthermore, for feasible conjugation, the SDF file format was converted to PDB format through the use of OpenBable (O'Boyle et al., 2011), and the atom types were manually verified. The oxime linker, which is one of the crucial components for linking the peptide and drug, was built, and the SMILES was retrieved and submitted to the OPSIN SMILES-to-PDB converter pipeline thereafter (Lowe et al., 2011). RDKit (Bento et al., 2020) was used to generate the coordinates through the platform’s distance geometry algorithm.

Finally, the peptide, oxime linker, and the apalutamide ligand were assembled into a single PDB, which represented a covalent conjugate. The assembly was done through the use of custom Python script, which employed packages such as the Biopython structure module v1.81 (Cock et al., 2009). To carry out this process, the chain identifiers were assigned, wherein Chain A represented the peptide, Chain B represented the oxime linker, and Chain C represented apalutamide (APL). The first bond was formed between the peptide’s N terminus and the oxime linker’s aldehyde carbon through covalent tethering with a bond distance of 1.47 Å. The second bond was formed between the oxime oxygen and the apalutamide’s thioamide nitrogen at a bond distance of 1.40 Å. This strategy is widely employed for peptide drug construct preparation. Furthermore, the construct was optimized through energy minimization for 5000 steps of steepest decent, translating to 0.01ns of system minimization through use of the GROMACS platform in a solvated environment (Abraham et al., 2015).

### 2.10 Molecular dynamic simulation

Molecular dynamics simulation serves as a pivotal tool for evaluating the stability of the ligand in the protein binding domain; it provides insights regarding the conformational changes taking place in the protein upon binding to the ligand (Hollingsworth and Dror, 2018). Additionally, it provides an idea about the minute rotameric changes taking place during the course of the simulation, resulting in dynamic behavior of the protein upon engagement with the ligand. The NiV G—CID24872560 complex was subjected to a molecular dynamics simulation of approximately 300 ns. The input file for the complex was prepared using the server-based input file generation server CHARMM-GUI (Jo et al., 2008). The ligand topology for CID24872560 was generated using the CGenFF ligand parameter generation module, which is integrated with the CHARMM-GUI server (Vanommeslaeghe et al., 2010; Kim et al., 2017). The simulation was carried out using the CHARMM36 force field. The initial energy minimization was carried out by executing 5,000 n steps of steepest descent in vacuum. Followed by this, the protein–ligand complex was subjected to solvation in a cubic periodic box with 0.5 nM buffer using the SPC water model (Qi et al., 2014). For replication of the local environment scenario for equilibrated dynamics, a neutral system was constructed with solvation of 0.15 M of Na^+^ and Cl^−^ ions. Concurrently, a short-scale production run was executed to equilibrate the system under standard NPT with 125,000 steps. The equilibration was performed while maintaining 1.0 bar of pressure and standard temperature of 303.15 K. For the designed cubical box simulation system, periodic boundary conditions were applied. Upon completion of the production run trajectory, a total of 3,000 frames containing different conformations were obtained. Subsequently, the evaluation of the data obtained from the simulated trace was carried out via the utilization of the XMGRACE package in GROMACS (Abraham et al., 2015). Parameters such as root mean square deviation (RMSD), root mean square fluctuation (RMSF), solvent accessible surface area (SASA), and the frequency of formed hydrogen bonds were evaluated. Furthermore, the investigation of the critical bonds that bring about the conformational changes was performed.

Additionally, to robustly assess the firmness and stability of the binding dynamics of the NiV G-CID24872560 complex and to address the gap of potential sampling bias, which may arise from the kinetic trapping within local energy minima, the complex was subjected to replicate molecular dynamics simulations with an independent set of initial atomic velocities (Schaffler et al., 2025). The parameters for the replicates were generated stochastically from the Maxwell–Boltzmann distribution at the target temperature (303.15 K) through the use of a randomized seed (gen seed = −1) (Huang and MacKerell, 2013; Büchel et al., 2021), which ensures that the replicate production trajectories diverges into distinct dynamical pathways from the same initial homologous starting conformation. This ensemble-based approach enables quantification of inter replicate variability, validation of convergence through cross comparison of the trajectories, and provides statistical robustness for all the derived observables, including RMSD, RMSF, SASA, and hydrogen bonding pattern estimates. The replicates were executed under identical NPT ensemble conditions (303.15 K, 1.0 bar) using the CHARMM36 force field and GROMACS, with each trajectory yielding approximately 3,000 frames for subsequent downstream analysis.

### 2.11 Binding free energy calculation through utilization of MMPBSA

For quantification of binding affinity between the NiV G and the selected immunogenic ligands, molecular mechanics Poisson–Boltzmann surface area (MMPBSA) analysis was performed. This end-state energy method integrates molecular mechanics with that of implicit solvation models to provide a detailed energetic profile of the protein–ligand complex. The calculations were performed with the aid of the gmxMMPBSA package integrated within the GROMACS software Package (Valdés-Tresanco et al., 2021).

The total binding energy free energy calculation was calculated using the equation:

$$\Delta G_{bind}=\Delta G_{solvation}\left(\;\Delta G_{SA}+\Delta G_{GB}\right)+\Delta G_{potential\;}\left(\Delta E_{ele}+\Delta E_{vdw}\right).$$

Herein, ***ΔE***_***vdw***_ and ***ΔE***_***ele***_ represent the van der Waals and the electrostatic interaction energies, respectively. Followed by ***ΔG***_***solvation***_, which encompasses the covalent (***ΔG***_***GB***_) and non-covalent (***ΔG***_***SA***_) desolvation components.

Complementary to this, per-residue decomposition was also evaluated through the use of the total decomposition contribution method to identify individual amino acids contributing significantly to the stability of the binding pocket. Additionally, it provides the level of binding strength between the ligand and the residues of interest and the overall stability of the complex.

## 3 Results

### 3.1 Finding the minimal epitope areas and testing their antigenicity, allergenicity, toxicity, and physicochemical characteristics

The SVM trip server was used to find the MER of the MMR vaccine, which was used to make the PLC more effective. The server provided six MERs for each vaccine, which fell within the accepted range of 0.9–1.0, as shown in Tables 1–3. We selected two MERs from each MMR vaccine because of their higher scores. The higher the score is, the higher the immunogenicity will be (Yao et al., 2012). After thoroughly examining the MER, we conducted additional assessments for their antigenicity, toxicity, and allergenicity, as shown in Table 5. We looked for the wet lab paper and found that the MMR vaccine induces a high antibody titer and long-term immunity (Mufson et al., 2015). According to the published study, the MER should be a 15- to 20-amino-acid sequence for effective action (Gershoni et al., 2007). Peptides within this range are optimal for transport across biological barriers and possess favorable pharmacokinetic properties (Klepach et al., 2022). All six MER underwent additional analysis using the ProtParam server for their PI, which unexpectedly fell within the range indicating that they would not precipitate in the blood. Table 4 shows all of the physicochemical parameters.

**TABLE 1:** Epitope prediction of the measles toxin using the SVMTrip server.

Rank	Location	Epitope	Score
1	520–539	QDLQYVLATYDTSRVEHAVV	1.000
2	461–480	NLALGVINTLEWIPRFKVSP	0.946
3	291–310	LGELKLAALCHGEDSITIPY	0.945
4	571–590	FTWDQKLWCRHFCVLADSES	0.941
5	81–100	TNSIEHQVKDVLTPLFKIIG	0.940
6	131–150	YDFRDLTWCINPPERIKLDY	0.940

**TABLE 2:** Epitope prediction of the mumps toxin using the SVMTrip server.

Rank	Location	Epitope	Score
1	226–245	SLLGSMTPAVVQATLSTSIS	1.000
2	423–442	VSLSNITYAENLTISLSQTI	0.784
3	260–279	VSVLLDEMQMIVKINVPTIV	0.715
4	335–354	YNEAERLSLETKLSVAGNIS	0.698
5	448–467	DISTELSKVNASLQNAVKYI	0.605
6	121–140	VTAAVSLVQAQTNARAIAAM	0.513

**TABLE 3:** Epitope prediction of the rubella toxin using the SVMTrip server.

Rank	Location	Epitope	Score
1	653–672	CARIWNGTQRACTFWAVNAY	1.000
2	259–278	PHTTERIETRSARHPWRIRF	0.997
3	739–758	VKFHTETRTVWQLSVAGVSC	0.997
4	850–869	APGPGEVWVTPVIGSQARKC	0.995
5	798–817	SRWGLGSPNCHGPDWASPVC	0.994
6	127–146	NPFQAAVARGLRPPLHDPDT	0.994

**TABLE 4:** Analysis of the physicochemical parameters of the eight minimal epitope regions of measles, mumps, and rubella toxins.

S.No	Minimal epitope regions of the vaccine	Vaccine	Mw (500–5,000 Da)	Instability index (≤40)	Aliphatic index	GRAVY (− values = hydrophilic; + values = hydrophobic)	Theoretical PI (>7.35–7.45<)	Half-life
1	QDLQYVLATYDTSRVEHAVV	Measles	2,307.55	41.84	107.00	−0.100	4.54	0.8 h (mammalian reticulocytes, *in vitro*) >10 h (yeast, *in vivo*) 10 h (*Escherichia coli*, *in vivo*)
2	NLALGVINTLEWIPRFKVSP	Measles	2,267.70	2.74	131.50	0.425	8.75	1.4 h (mammalian reticulocytes, *in vitro*) 3 min (yeast, *in vivo*) >10 h (*Escherichia coli*, *in vivo*)
3	SLLGSMTPAVVQATLSTSIS	Mumps	1,963.27	17.69	117.00	0.910	5.24	1.9 h (mammalian reticulocytes, *in vitro*) >20 h (yeast, *in vivo*) >10 h (*Escherichia coli*, *in vivo*)
4	VSLSNITYAENLTISLSQTI	Mumps	2,167.44	52.94	136.50	0.515	4.00	100 h (mammalian reticulocytes, *in vitro*) >20 h (yeast, *in vivo*) >10 h (*Escherichia coli*, *in vivo*)
5	CARIWNGTQRACTFWAVNAY	Rubella	2,331.65	37.06	54.00	0.035	8.96	1.2 h (mammalian reticulocytes, *in vitro*) >20 h (yeast, *in vivo*) >10 h (*Escherichia coli*, *in vivo*)
6	PHTTERIETRSARHPWRIRF	Rubella	2,546.88	66.05	44.00	−1.465	11.82	>20 h (mammalian reticulocytes, *in vitro*) >20 h (yeast, *in vivo*)

The allergenicity, toxicity, and antigenicity profiles of six MERs were checked through their respective servers, which are shown in Table 5. It was discovered that none of these peptides was harmful or caused allergies. The six MERs were found based on their toxicity, allergenicity, and physicochemical properties. To show that these MERs are stable and can trigger an immune response without harming the host, the values must be within the acceptable ranges. We selected the two most suitable minimum epitopes, comprising one mumps MER (VSLSNITYAENLTISLSQTI) and one rubella MER (PHTTERIETRSARHPWRIRF), for PLC design targeting NiV G. We chose these above two MERs because of their high average half-life and fall under all the parameters. Furthermore, we have investigated possible T-cell epitope-binding by our previous two selected peptides using NetMHC (https://services.healthtech.dtu.dk/services/NetMHC-4.0/) and found that mumps MER (VSLSNITYAENLTISLSQTI) has strong binding 9-MER core epitope (LSNITYAEI) within the peptide sequence VSLSNITYAENLTISLSQTI for the HLA-A*01:01* allele (%Rank = 0.40), indicating potential CD8^+^ T-cell immunogenicity and can trigger the immune response. Similarly, for MHC class II (https://services.healthtech.dtu.dk/services/NetMHCII-2.3/) binding analysis revealed that the peptide VSLSNITYAENLTISLSQTI exhibited strong binding affinity toward HLA-DRB1*04:01 (IC50 = 88.7 nM; %Rank = 1.40). The core epitope YAENLTISL was identified as the primary binding region, indicating the potential to elicit CD4^+^ T-cell-mediated immune responses. For rubella MER (PHTTERIETRSARHPWRIRF) there is no such T-cell response is observed; we chose this peptide due to its good physiochemical parameters.

**TABLE 5:** Antigenicity, toxicity, and allergenicity profile of MER of MMR vaccine.

S. no	Minimal epitope regions	Vaccine	Toxicity	Allergenicity (non-allergen)	Antigenicity
1	QDLQYVLATYDTSRVEHAVV	Measles	Non-toxic	Non-allergen	0.6089
2	NLALGVINTLEWIPRFKVSP	Measles	Non-toxic	Probable allergen	1.1238
3	SLLGSMTPAVVQATLSTSIS	Mumps	Non-toxic	Non-allergen	0.5557
4	VSLSNITYAENLTISLSQTI	Mumps	Non-toxic	Non-allergen	1.0283
5	CARIWNGTQRACTFWAVNAY	Rubella	Non-toxic	Non-allergen	Non-antigen −0.1682
6	PHTTERIETRSARHPWRIRF	Rubella	Non-toxic	Non-allergen	0.9249

### 3.2 ADME analysis of hit compound

All screened compounds exhibited molecular weights ranging from 317.2 to 477.0 Da, which fall within the acceptable range (<500 Da) for orally active drugs. The predicted LogP values (1.418–4.178) indicate moderate lipophilicity, suggesting a favorable balance between aqueous solubility and membrane permeability, as shown in Table 6. Importantly, none of the compounds showed any Lipinski rule violations, supporting their potential oral bioavailability.

**TABLE 6:** ADME analysis of the top 10 selected compounds.

S.no	CID	MW (100–600)	LogP At pH = 7.4	Lipinski violation	BBB (0–1)	CYP3A4 Inh (0–1)	hERG (0–1)	HLM stability (0–1)	*T*_1/2_
1	24872560	477.09	3.201	0.0	0.002	0.992	0.393	0.31	1.179
2	48229	414.3	4.138	0.0	0.95734	0.03607	0.22	0.999167	0.77
3	25273599	414.3	4.178	0.0	0.599284	0.032436	0.717083	1.0	0.997
4	6918248	390.4	3.816	0.0	0.959	0.0	0.834	0.33	0.252
5	76958517	406.4	3.516	0.0	0.479	1.0	0.247	0.997	0.865
6	11610526	377.14	2.503	0	0.992	0.002	0.922	0.129	2.194
7	3,168	379.4	3.565	0	0.976	0.001	0.898	0.871	0.223
8	24905400	387.9	387.9	0	0.409	0.762	0.629	0.766	0.215
9	72,106	395.4	3.979	0	0.435	0.251	0.65	0.483	0.411
10	4,493	317.22	1.418	0	0.0	0.104	0.046	0.157	1.032

Collectively, the ADME assessment suggests that the selected compounds possess favorable pharmacokinetic characteristics, with manageable metabolic and toxicity risks, warranting further structural optimization and experimental validation.

### 3.3 Molecular docking study

We used molecular docking on 50 FDA-approved compounds to find the best ones to use in making the PLC. We looked at all the important docking parameters and did site-specific docking to find all the possible ligand–protein interaction sites. The exhaustiveness value was set to 8, which meant that each ligand was docked eight times to make sure the scores were accurate. There were 400 different docking poses generated in all. The ligands were screened and filtered throughout the process to arrive at 10 ligands that had higher docking scores than the control compound. The top 10 ligands were selected and designed for the PLC for the NiV G protein. The ligand with the best docking score was CID24872560, which had a docking score of −9.7 kcal/mol. Based on its docking score, it would show the greatest binding affinity for the target NiV G and was determined to fit best within the binding pocket of this protein. Table 7 shows the top 10 hits against the NiV G. To further enhance the robustness, we employed docking protocols for the complete PLC predicted construct with the NiV G protein. For the execution of this task, the system-installable platform AutoDock Vina was used, so as to carry out the docking procedure, the grid box dimensions were set to 20 × 20 × 20 Å, where the grid extended to ±10 Å toward each edge. The grid box was centered at coordinates (32.57, 60.01, and 35.22), which coincide with the center of the ephrinB2 binding site. The selected position of the grid box contained all 20 critical residues of NiV G as reported in PDB 2VSM (Bowden et al., 2008). The assigned dimension of the grid box allowed sufficient sampling space for the predicted PLC construct while maintaining computational traceability. Furthermore, the exhaustive parameter was set to 16 to balance the search efficiency and computational accuracy.

**TABLE 7:** Top 10 docking hits against Nipah virus glycoprotein.

S.no.	CID (ligand)	IUPAC name	Docking score (kcal/mol)
1	24872560	4-[7-[6-Cyano-5-(trifluoromethyl)-3-pyridinyl]-8-oxo-6-sulfanylidene-5,7-diazaspiro[3.4]octan-5-yl]-2-fluoro-N-methylbenzamide	−9.7
2	48,229	(3-Oxo-1H-2-benzofuran-1-yl) 2-[3-(trifluoromethyl)anilino]pyridine-3-carboxylate	−9.2
3	25273599	[(1R)-3-oxo-1H-2-benzofuran-1-yl] 2-[3-(trifluoromethyl)anilino]pyridine-3-carboxylate	−9.1
4	6918248	3-[2-[4-[3-(Trifluoromethyl)phenyl]piperazin-1-yl]ethyl]-1H-benzimidazol-2-one	−9
5	76958517	[(2R)-2,3-dihydroxypropyl] 2-[[8-(trifluoromethyl)quinolin-4-yl]amino]benzoate	−9
6	11610526	2,4,6-Trifluoro-N-[6-(1-methylpiperidine-4-carbonyl)-2-pyridinyl]benzamide	−8.9
7	3,168	3-[1-[4-(4-Fluorophenyl)-4-oxobutyl]-3,6-dihydro-2H-pyridin-4-yl]-1H-benzimidazol-2-one	−8.6
8	24905400	4-(2-Chlorophenoxy)-N-[3-(methylcarbamoyl)phenyl]piperidine-1-carboxamide	−8.3
9	72,106	2-Morpholin-4-ylethyl 2-[3-(trifluoromethyl)anilino]pyridine-3-carboxylate	−8.2
10	4,493	5,5-Dimethyl-3-[4-nitro-3-(trifluoromethyl)phenyl]imidazolidine-2,4-dione	−8.1
Positive control (tecovirimat) protease complex inhibitor			
1	16124688	N-[(1R,2R,6S,7S,8S,10R)-3,5-dioxo-4-azatetracyclo[5.3.2.02,6.08,10]dodec-11-en-4-yl]-4-(trifluoromethyl)benzamide	−8.17

### 3.4 Molecular interaction study of NiV G and its targeted drug

**FIGURE 2:**
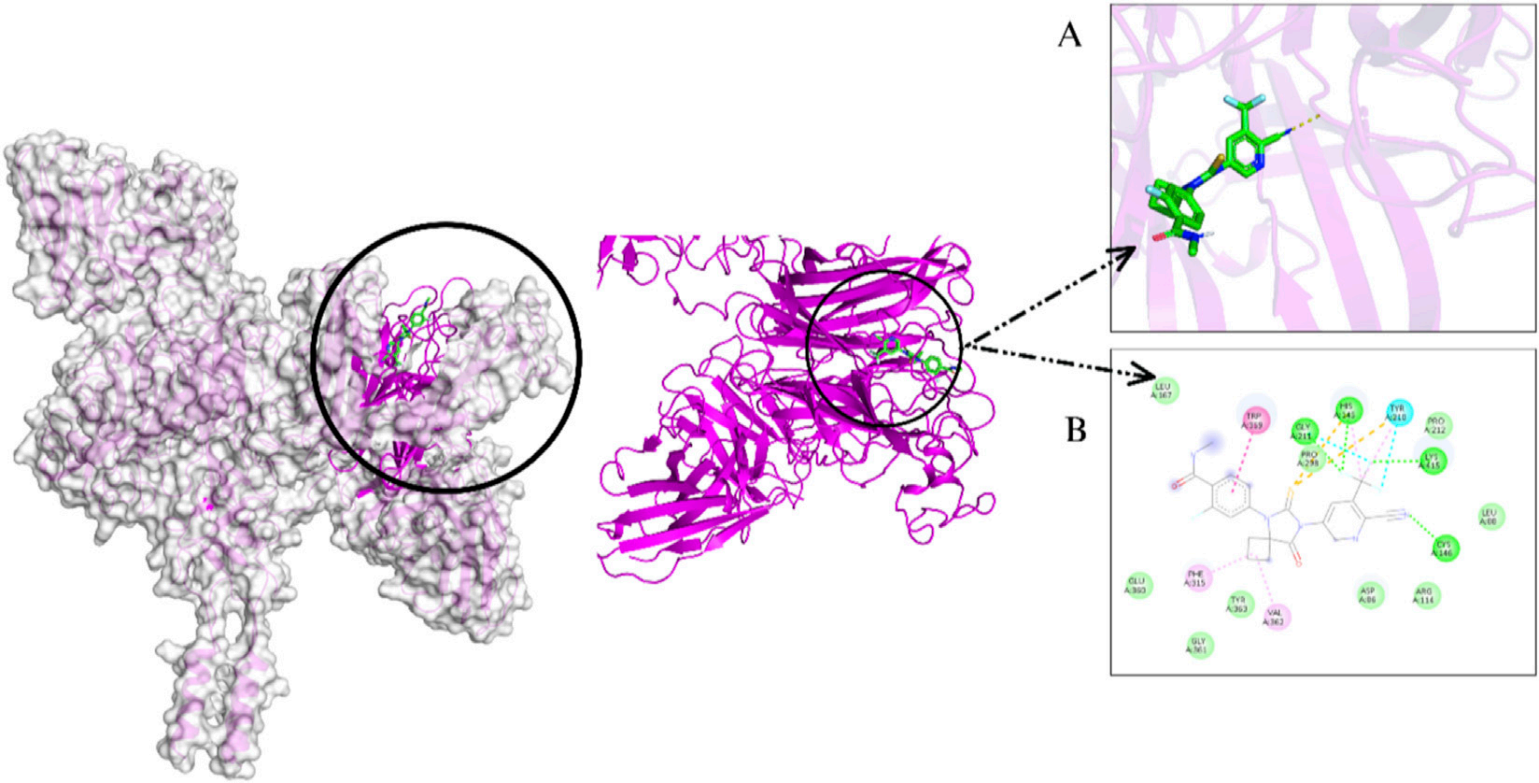
This shows how the ligand binds to the surface of the active site pocket of the Nipah G protein. **(A)** Molecular interaction between protein and ligand via bond formation in three-dimensional orientation. **(B)** 2D map of protein–ligand interactions.

After looking at the top ten hits and their docking scores, one compound (PubChem ID24872560) was chosen for in-depth molecular interaction studies with its target protein. Apalutamide is classified as a non-steroidal anti-androgen. It is structurally a spirocyclic compound with an attached trifluoromethyl pyridine and an amide- or thio-containing moiety, making it structurally stable, capable of binding well to androgen receptors in clinical practice, and it has been used previously for drug development. Apalutamide received marketing approval from the FDA in 2018 (https;//newdrugapprovals.org/tag/apalutamide/) as a validated therapy for metastatic castration-resistant prostate cancer (mCRPC). We selected this drug because it demonstrated significant efficacy against Severe Acute Respiratory Syndrome Coronavirus 2 (Majidipur et al., 2023). Although it is not currently recommended as a standard antiviral therapy, previous studies have reported promising antiviral activity against SARS-CoV-2 (Majidipur et al., 2023). Figure 2 shows how it interacts with the Nipah glycoprotein at the molecular level. PyMOL and CLC drug discovery visualizer software (Rigsby and Parker, 2016; Biovia, 2020) were used to generate the molecular interaction diagrams. Extensive interaction studies (see Figure 2B) showed that the ligand had a number of conventional hydrogen bonds with important amino acids, His145, Gly211, Lys415, and Cys146 within the catalytic site. In addition to the five hydrogen bonds, there were also several hydrophobic interactions (e.g., π-alkyl interactions, π–π stacking interactions, and van der Waals interactions) that contributed to the stability of the complex. In particular, the hydrophobic residues such as Trp359, Phe315, and Val362, along with other surrounding hydrophobic residues, were important contributor to ligand-binding stability. Molecular docking studies on the target NiV G glycoprotein identified some predicted binding sites that do not overlap with the binding interface of the host receptors (ephrin-B2/B3). In particular, the NiV G protein interacts with four critical residues located at the tip of the G–H loop of ephrin-B2/B3 (Trp125, Leu124, Pro122, and Tyr/Phe120), which insert into four preformed hydrophobic pockets in the G protein cavity (Bowden et al., 2008). In addition, Glu533 of NiV G forms two important salt bridges with Arg57 and Lys116 of ephrin, and mutations at Glu533 have been shown to completely abolish viral attachment and membrane fusion (Guillaume et al., 2006; Negrete et al., 2007). Our docking analysis shows that the selected drug binds to a different regions of the NiV G protein than the region where the ephrin-B2/B3 binds.

### 3.5 Molecular docking on glycosylated NiV G protein

We re-performed molecular docking using the fully glycosylated NiV G structure without removing the glycan moieties. Compared with the deglycosylated model, the ligand exhibited a substantially reduced docking score of −4.3 kcal/mol and did not form any hydrogen bond interactions with the protein. This difference in binding affinity was likely due to the steric hindrance from the attached glycans that prevented the ligand from entering the binding pocket and changed the orientation and interaction dynamics of the complex. For this reason, a de-glycosylated model was used to perform a detailed interaction analysis and to run the simulations presented in this manuscript. In addition, the docking results for NiV G demonstrate how the glycans on the surface of the protein may modulate ligand-binding, indicating that glycosylation should be considered in all future studies to better evaluate the physiological relevance of the proposed peptide–ligand conjugates. The detailed figure and associated interactions are presented in Supplementary Figure S1.

### 3.6 Molecular dynamic simulation revelations

**FIGURE 3:**
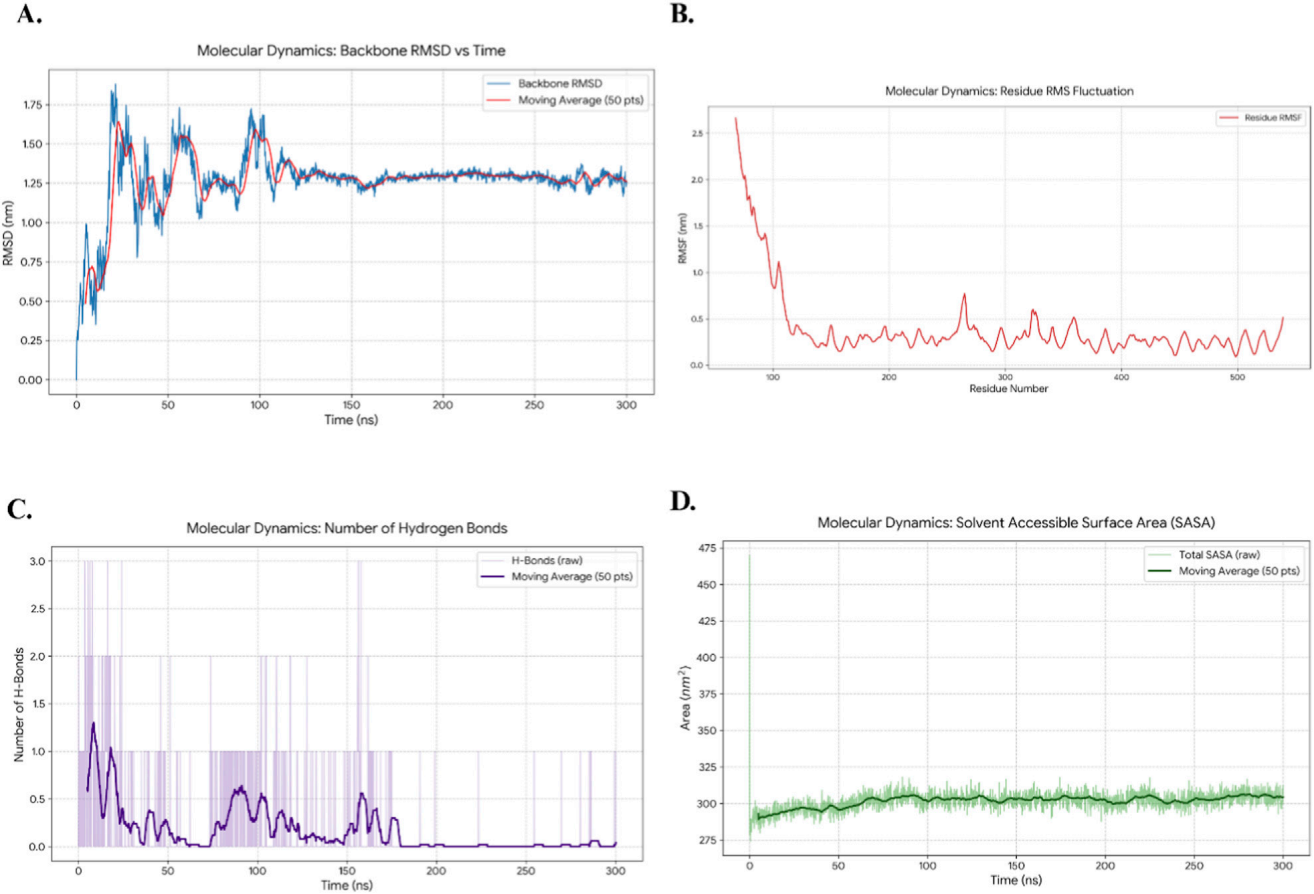
Molecular dynamic simulation of glycoprotein—CID24872560, representing **(A)** RMSD, **(B)** RMSF, **(C)** the number of hydrogen bonds, and **(D)** SASA.

Molecular dynamic simulation plays a pivotal role in augmenting the results derived outputs from molecular docking studies through a virtual dynamic biological environment. This provides insight into critical interaction events occurring over a longer time frame and provides critical overviews on the flexibility and volatility of the host protein and the engaged ligand. In our study, a 300-ns molecular dynamic simulation was performed by utilizing the GROMACS open-source simulation platform. For the simulation, our study proceeded with CID24872560-NiV G complex, which showcased the best binding affinity during the molecular docking studies, were further subjected to molecular dynamic simulation studies, spanning 300 ns. The outcomes retrieved from the production run showcased phenomenal trajectories upon root mean square deviation analysis; the average deviation observed was calculated to be approximately 1.26 Å, where the initial peak represented in the RMSD trace accounts for the high dynamicity in the residues located in the neck region, which forms a series of beta turns, thereby connecting the stalk to the beta sheet bundles. Furthermore, it was observed that in the later stage of the simulation, the whole complex was observed to be stabilized, with a maximal deviation of 1.7 Å and minima of 1.13 Å after 100 ns of simulation, which indicates the retention of the destined critical ligand pose in complement with the protein as showcased in Figure 3A. A similar trend was also reflected in the root mean square fluctuation profiles for the simulated complex where those residues that were in non-bound regions particularly in the neck region of the protein showed a higher RMSF compared with residues which were involved in formation of the beta sheet bundles, similar to the residues in the beta sheet bundle, the residues which were interacting with the ligand CID24872560 showed a similar trend with lower RMSF thus conferring stability and firmness to the complex. The RMSF profiles further inferred that the residues that were involved in the main structure framework formation for the NiV G head showed minimal deviation from the initial structure with simultaneous retention of the ligand in the binding pocket as shown in Figure 3B. The hydrogen plot depicted maximal polar hydrogen interactions of about 3 and with minimal of at least 1, a consistent retention of polar interaction was observed for majority of the production run especially with Gly289, His218, Lys497, and Cys219 upon visualization of the simulated frames a mix of polar hydrogen and non-polar hydrophobic Pi–Pi and Pi–cation stacking interaction, which are crucial for hold the ligand in its binding domain like those with Trp 441 was observed. Predominantly, the polar contacts were observed to be dominant, thus not allowing any disengagement from the binding pocket as depicted in Figure 3B. The analysis further proceeded to the calculation of solvent accessible surface area, which provides us with the information regarding the surface area available for any other ligand to engage with the binding pocket; hence, the lower the SASA, the lower the chances of any other secondary ligand to come and engage. From the SASA plot, it was inferred that the maximum available surface area left free was found to be 310 nm^2^ as via inferred via Figure 3D. Simultaneously, it indicated a very compact and strong engagement between the NiV G head and the ligand—CID24872560. Additionally, the average leftover SASA space of 310 nm^2^ provides an ideal microenvironmental area for stable, specific and immunologically effective peptide–ligand conjugate deployment. Complementing these outcomes, replicate with different initial velocities and induced seeds was run, the RMSD backbone showcased a robust convergence and consistent sampling behavior, the elevated RMSD in the initial stages toward the end reflected subtle conformational adjustment in the peripheral loop regions without compromising the binding pocket behavior. However, the RMSF provided intact behavior of residual changes, which inferred a geometric and dynamic sturdiness across different initial velocity conditions. Upon calculation of SASA, it displayed homology to that of the default conditional simulation behavior with an average of 300 nm^2^ of available solvent assessable surface profile. Hence, supporting the notion of proper burial of the peptide epitope tethered with the linker and compound. A similar trend was also reflected in the case of hydrogen bond retention analysis, with consistent interaction along some brief drops post 200 ns and further enhanced polar contacts post 250 ns, as reflected in the Supplementary Figure S2.

### 3.7 MMPBSA analysis revelations

The thermodynamic stability and the binding affinity for the NiV G in complex with the immunogenic ligand CID24872560 were quantified through the use of the MMPBSA method. The revelations from this analysis provided the study with an intricate breakdown analysis of the energetic components contributing to the overall binding free energy (***ΔG***_***bind***_).

The binding poses were characterized by highly favorable non-covalent interactions. The displayed output average for the weak-bond van der Waals energy (***ΔE***_***vwd***_) was measured to be −1,648.81 kcal/mol; this suggested a steadfast complementary fit within the ligand’s spirocyclic scaffold and the hydrophobic residues belonging to the glycoprotein’s binding pocket. The determined mean of the electrostatic energy (***ΔE***_***ele***_) was observed to have an average contribution of −14,220.04 kcal/mol; this indicated robust polar interactions and hydrogen bonding.

Complementing this data, per-residue energy decomposition was also measured; this aids in the identification of critical amino acid residues that govern the recognition and stabilization of the ligand in the canonical pocket. This analysis unveiled multiple hotspot residues that are involved in disproportionate contribution to the ligand-binding stability.

**FIGURE 4:**
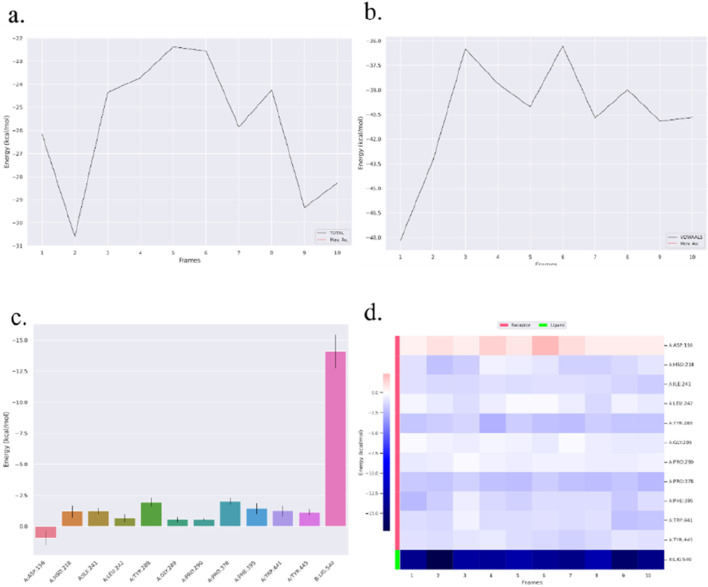
The figure illustrates the energetics-based outcomes derived from MMPBSA analysis. **(a)** Illustration of fluctuation trace throughout the simulation for overall ΔGbind (delta Gibbs free energy), inferring toward overall stability of the complex. **(b)** This frame displays the variations taking place in overall non-polar interactions throughout the course of the simulation. **(c)** Representation of critical residue-wise contacts retained throughout the production run alongside corroborated total energy for the ligand. **(d)** Temporal evolution heat map for per-residue energy decomposition, which illustrates the binding free energy contributions for individual critical residues. Blue color indicates energetically favorable residue contributions, while lighter shades represent neutral or destabilizing interactions.

The top residues that displayed the greatest binding contribution were observed to be Arg185, demonstrating the highest contribution, which showcased an average energy of −105.85 kcal/mol; this acted as the primary anchor point through strong electrostatic interactions. Similarly, the Asp156 was observed to contribute significantly with a mean energy of −31.17 kcal/mol, which is more likely to have been involved in the formation of a salt bridge or some key polar contact with the ligand’s amide moiety. Gln496 and Lys497 were observed with significant contributions of −14.03 and −13.10 kcal/mol, respectively; this further secures the ligand within the orthosteric domain. In addition to this, some of the hydrophobic involvements were also observed with the residues like those of Leu158 and Leu242. This inference supplements the structural integrity of the complex throughout the 300 ns trajectory. All the derived MMPBSA outcomes has been collated in Figure 4.

### 3.8 Carbon skeleton of peptide–ligand conjugates using a suitable linker

**FIGURE 5:**
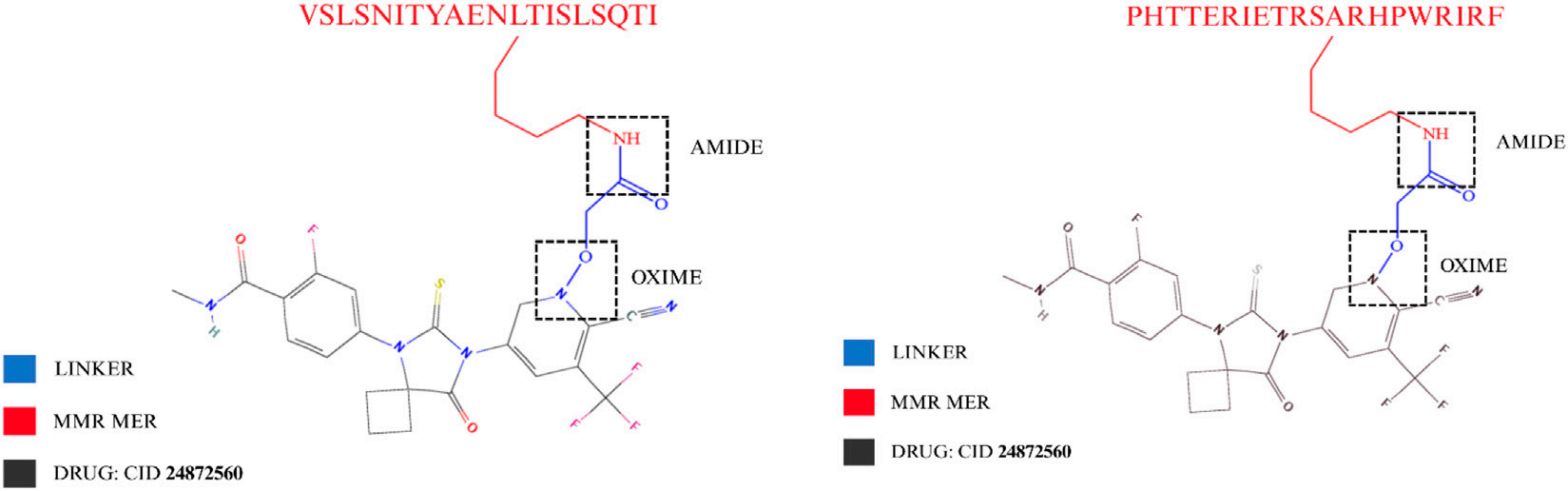
Carbon skeleton representation of the peptide–ligand conjugate constructed using an oxime linker. The minimal epitope region (MER) is highlighted in red, whereas the oxime linker is shown in blue, with the newly formed bond indicating successful conjugation. The drug moiety, depicted in black, corresponds to the compound with the highest docking score, illustrating its stable attachment to the peptide through the non-cleavable linker.

The linker was designed to be non-cleavable, ensuring stable attachment between the ligand and the peptide for enhanced biological activity. An oxime linker was selected as a suitable candidate because it enables efficient conjugation through the free carbonyl group of the ligand, while forming a stable amide bond with the selected peptide, as illustrated in Figure 5. Two MERs—the mumps MER (VSLSNITYAENLTISLSQTI) and the rubella MER (PHTTERIETRSARHPWRIRF)—were conjugated with the highest-scoring compound hits. The conjugation process was carried out using ChemBioDraw Ultra 14.0 (https://chembiodraw-ultra.software.informer.com/), allowing precise structural modeling of the peptide–ligand conjugates.

**FIGURE 6:**
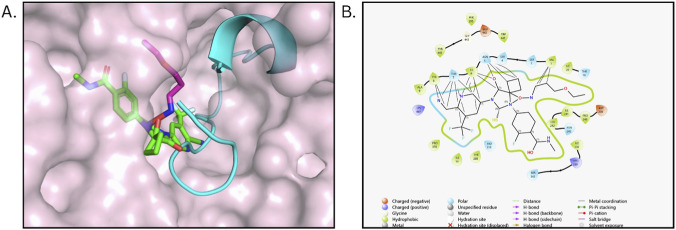
**(A)** The structure of the peptide–ligand conjugate (PLC) complexed with the Nipah virus (NiV) G Glycoprotein is presented above. The NiV G protein is depicted using a light violet color surface model, whereas the ligand is depicted using a green surface model, occupying the binding pocket and extending from an oxime-linked (pink color) MMR-derived minimal epitope peptide (light blue color) to the solvent accessible area, indicating potential accessibility to antibodies. **(B)** 2D interaction diagram of the PLC bound to the NiV G glycoprotein, highlighting extensive polar interactions between the ligand moiety and key residues within the binding pocket.

### 3.9 Structural validation of the ligand–linker–peptide architecture

The entire combination of peptide, ligand, and linkers was examined as the complete conjugate modelled by 3D computer modeling using the NiV G protein as the tractable target site for generating these peptide ligands against the virus. Structural analysis and molecular dynamics simulations confirmed that although the ligand remained attached within the binding pocket, the linkers positioned the peptide in a conformation, where it was accessible to circulating antibodies, allowing relatively low steric hindrance as reflected in Figure 6.

## 4 Discussion

Vaccination induces adaptive immunity, which has proven to be one of the most effective strategies for preventing infectious diseases. A key advantage of adaptive immunity is its antigen-specific response, enabling precise recognition and elimination of pathogens. This immune protection is mediated through the activation and expansion of B and T lymphocytes, which play essential roles in antibody production, immune memory formation, and pathogen clearance. Despite significant technological advances, there has been a rising trend in NiV infection (Singh et al., 2019). This shows that a more innovative and alternative approach is required to treat NiV infection. Despite of this, there are no approved antiviral treatments or vaccines available (De and Clercq, 2001). Hence, the current work opted for an innovative peptide–ligand conjugate–based immunotherapeutic approach to treat NiV infection. The therapeutic target of the NiV is the glycoprotein (G). The G protein attaches to the host cell receptors (ephrin-B2/B3). This G protein is a critical target for vaccine development and therapeutic antibodies due to its essential role in viral entry and high pathogenicity (Xu et al., 2012; Ortega et al., 2022).

In this study, we designed a PLC-based immunotherapeutic approach that can target the NiV-derived G protein, allowing the elimination of both substances. The drugs were downloaded from the MolProphet database, and the 50 drugs that are FDA-approved were docked against the G protein, with the four top hits selected for use as inhibitor compounds during the experiments. To determine which of the four compounds would have the best possible effect, the ADME data for the inhibitors were assessed, and the top-ranked hit was selected for the simulation portion of the study. The molecular docking and simulation procedure represents a preliminary approach to discovering new lead molecules for the treatment of many different viruses, including those that cause antiviral infections. There are concerns with the off-target effects of the proposed PLC-based immunotherapies, particularly with apalutamide, which has been shown to have potential antiviral effects against SARS-CoV-2 (Majidipur et al., 2023) and should not be used concomitantly with other medications due to potential drug–drug interactions. The compounds that exhibit activity against the above-mentioned G protein target will do so through their individual actions as individual compounds. Our proposed PLC, composed of a massive arrangement of FDA-approved drugs, a non-cleavable linker, and an immunogenic peptide, upon injection into the bloodstream, will lose the ability to cross cell membranes but will ultimately have selective affinity toward the G protein; therefore, it is also designed to be used as a treatment modality.

All of the ligands listed above have much greater binding affinities than the control ligand; they could also potentially bind to and help prevent G protein binding to ephrin-B2/B3. This could lead to inhibition and activation of the complement pathway, ADCC, and macrophages in a manner that would also non-specifically inhibit NiV G. At the same time, these drugs may target all of the PLCs for the purpose of inducing immune responses to eliminate them. For this purpose, we chose to use epitopes from the MMR vaccine and have further evaluated the MMR vaccine epitopes using *in silico* methods to determine their antigenicity, allergenicity, and toxicity profiles. The ProtParam server was utilized to identify the half-lives and stability indices of stably selected peptides. The immune response generated by a peptide epitope should stimulate pre-existing vaccine-induced memory T- and B-cell responses through a quick immune response that effectively eliminates the G protein from the body. MMR vaccines induce lifelong immunity and generate high levels of antibody titers; there should be sufficient antibodies to generate ADCC-mediated elimination of NiV G.

A non-cleavable oxime linker was used to bind both the ligand and the peptide together, which will prevent the premature release of the peptide and ligand (Fu et al., 2023). We chose this type of linker because it does not to undergo premature cleavage in plasma, unlike other linkers, before reaching the target, thereby reducing off-target toxicity while maintaining high stability under different pH conditions.

The peptide for therapeutic use may have some limitations, such as low bioavailability, limited stability, and solubility issues in the body system, so further research is required for its biological feasibility. Peptide drug conjugates have widely succeeded in cancer therapy (Cooper et al., 2021); however, the peptide in our designed PLC has not been individually tested for any therapeutic purpose. Using a server-based structural modeling platform, we have successfully modeled the entire peptide–ligand conjugate (PLC) bound to the glycoprotein of NiV G and verified that the structure is geometrically feasible. The results also confirmed that the peptide was solvent-exposed and accessible for recognition by an antibody.

The proposed PLC has not yet been tested in NiV treatment, but similar immune-based therapy has been successfully experimented with in influenza virus in *in vitro* and *in vivo* studies (Liu et al., 2020; Debroy et al., 2023).

The peptide-based vaccines (MMR) used in our study were sourced from the Nipah endemic countries where they have been shown to confer lifelong immunity (Johnson et al., 1996), ensuring that the resulting conjugation also offers long-term protection.

Although docking and simulation techniques have been utilized to assess the overall behavior of a PLC, there is a likelihood that PLCs will behave differently when subjected to a physiological environment compared with a simulated model as a result of various factors such as linker flexibility, peptide conformation, and steric hindrance. This research represents a rational basis for computational design of PLCs to target the NiV and may provide a starting point for future experimental validation and therapeutic development.

## 5 Conclusion

The *in silico* design of innovative PLCs aimed at the NiV attachment glycoprotein represents a promising approach for the management of severe NiV infections. This method, which uses a pre-existing immunotherapeutic approach, could also be used to target other viruses, making it a flexible and adaptable way to treat viral infections. Because there are no approved treatments for NiV, the PLC strategy approach offers a new way to treat the disease. This could result in greater selectivity and fewer side effects, potentially enhancing patient outcomes. However, our findings are entirely based on computational analyses. Substantial *in vitro* and *in vivo* experimentation is essential to validate the immunogenicity, safety, efficacy, and potential clinical applicability.

## Data Availability

The data supporting the findings of this study are available and can be accessed through the following Zenodo DOI - https://doi.org/10.5281/zenodo.20226225.
