# Supplementary material for: Computational design of immunogenic peptide–ligand conjugates for targeted therapy against Nipah virus infection
Source: Front Bioinform. 2026 Jul 27;6:1846380. doi: 10.3389/fbinf.2026.1846380 (PMC13454029; doi:10.3389/fbinf.2026.1846380)
Supplement: Supplementary file 1 [file Supplementaryfile1.docx]

**Supplementary file**

1. **Molecular docking on Glycosylated NiV G protein**

Supplementary Fig-1 Molecular docking of the ligand with the glycosylated Nipah virus G glycoprotein. The left panel shows the docked complex with the fully glycosylated NiV G protein represented as a surface model. The middle panel highlights the ligand-binding pocket (black circle) within the receptor-binding region. The right panel presents the two-dimensional interaction diagram, showing limited interactions of the ligand with residues Val300 and Asn306. The reduced number of contacts and absence of hydrogen bonds indicate that surface glycans restrict ligand accessibility and decrease binding affinity in the glycosylated model.

a.

b.

c.

d.

Supplementary Fig-2 Illustration of the trajectory traces derived from replicate simulation of Glycoprotein- CID24872560 with differed initial velocity.; a) representation of RMSD trace, representing the overall structural displacement from the initial conformation. b) Display of the RMSF plot, depicting the residue level deviation for protein ligand complex . This aids in assessment of tacking unit level deviation. c) Representation of Hydrogen Bond plot, provide the information regarding the number of formed and deformed polar contacts between the ligand and the protein. d) Display of SASA plot, provide the intel regard the available surface area which can be accessed by the solvent.

Video Illustration 1: The video displays the occupation of the Peptide Ligand Conjugate (PLC), in the binding domain of NiV G protein, showcasing its steadfast binding and dynamic stability.
